# Cachexia index as a prognostic predictor after resection of pancreatic ductal adenocarcinoma

**DOI:** 10.1002/ags3.12686

**Published:** 2023-04-24

**Authors:** Tomonari Shimagaki, Keishi Sugimachi, Yohei Mano, Emi Onishi, Tomohiro Iguchi, Yuichiro Nakashima, Masahiko Sugiyama, Manabu Yamamoto, Masaru Morita, Yasushi Toh

**Affiliations:** ^1^ Department of Hepatobiliary and Pancreatic Surgery National Hospital Organization Kyushu Cancer Center Fukuoka Japan; ^2^ Department of Gastroenterological Surgery National Hospital Organization Kyushu Cancer Center Fukuoka Japan

**Keywords:** cachexia index, pancreatic ductal adenocarcinoma, pancreatic resection

## Abstract

**Aim:**

This study was performed to investigate the relationship between the preoperative cachexia index (CXI) and long‐term outcomes in patients who have undergone radical resection of pancreatic ductal adenocarcinoma (PDAC).

**Methods:**

In total, 144 patients who underwent pancreatic resection for treatment of PDAC were retrospectively analyzed. The relationship between the CXI and the patients' long‐term outcomes after PDAC resection was investigated. The CXI was calculated based on the preoperative skeletal muscle index, serum albumin level, and neutrophil‐to‐lymphocyte ratio. After propensity‐score matching, we compared clinicopathological features and outcomes.

**Results:**

The multivariate analysis showed that lymph node metastasis (hazard ratio [HR], 1.93; 95% confidence interval [CI], 1.16–3.23; *P =* 0.0118), R1 resection (HR, 57.20; 95% CI, 9.39–348.30; *P <* 0.0001), and a low CXI (HR, 2.10; 95% CI, 1.27–3.46; *P =* 0.0038) were independent and significant predictors of disease‐free survival (DFS) after PDAC resection. Moreover, a low CXI (HR, 3.14; 95% CI, 1.71–5.75; *P =* 0.0002) was an independent and significant predictor of overall survival (OS) after PDAC resection. After propensity‐score matching, the low CXI group had a significantly worse prognosis than the high CXI group for both DFS and OS.

**Conclusion:**

The CXI can be a useful prognostic factor for DFS and OS after pancreatic resection for treatment of PDAC.

## Graphical abstract

The CXI can be a useful prognostic factor for disease‐free survival and overall survival after pancreatic resection for treatment of PDAC.

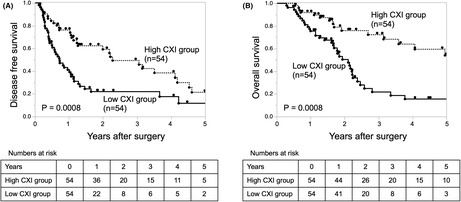

## 1 INTRODUCTION

Pancreatic ductal adenocarcinoma (PDAC) is one of the most lethal cancers worldwide because of its high malignant potential and the difficulty of detecting the cancer at an early stage.^1^ Surgical resection is the only curative option in patients with PDAC; however, the prognosis is poor.^2^ Therefore, it is important to identify a biomarker to optimize treatment and predict the prognosis for patients with PDAC.

Increasing evidence has recently shown that the nutritional and immunological status are independent poor prognostic factors in patients with liver, stomach, renal, and lung cancer.^3^, ^4^, ^5^, ^6^ As systemic inflammatory and immunological markers, the neutrophil‐to‐lymphocyte ratio (NLR) and platelet‐to‐lymphocyte ratio (PLR), which are based on blood cell counts, have been well studied. The prognostic nutritional index (PNI), Glasgow Prognostic Score, modified Glasgow Prognostic Score (mGPS), C‐reactive protein (CRP)‐to‐albumin ratio, and Controlling Nutritional Status (CONUT) score, which are based on the albumin level, are reportedly associated with surgical outcomes in patients undergoing surgical resection of PDAC.^7^, ^8^ However, those markers do not directly reflect the effect of sarcopenia associated with cancers.

Cachexia is a complex metabolic disorder characterized by muscle loss with or without loss of fat mass associated with the underlying disorder. Cachexia is associated with more than 50% of cancer deaths and causes death in ~30% of patients with cancer.^9^ The cachexia index (CXI), which consists of the skeletal muscle index (SMI), serum albumin level, and NLR, was recently established to comprehensively reflect the cachectic status.^10^ CXI is a new marker that directly includes all factors of sarcopenia, inflammation, and nutrition, but the impact of the CXI on the prognosis of PDAC has never been reported.

In this study we examined the possibility that the CXI could be a better prognostic factor for PDAC than sarcopenia and the NLR, which have already been reported as prognostic factors for PDAC.

## 2 METHODS

### 2.1 Study design

This retrospective study was conducted using prospectively collected and maintained data. The study included 144 consecutive patients with cytohistologically proven PDAC who underwent curative‐intent pancreatectomy from January 2014 to December 2021 at the Kyushu Cancer Center. The cases that resulted in nonresection were excluded from the analysis. In this study, we used the 7th edition of the classification of pancreatic carcinoma by the Japan Pancreas Society. When classified by initial resectability, 131 cases were classified as resectable, seven as borderline resectable with artery involvement, and six as borderline resectable with portal vein involvement (Table 1). Postoperative complications were evaluated based on the Clavien–Dindo classification.^11^

**TABLE 1:** Comparative analysis of clinicopathological parameters between the high‐CXI group and low‐CXI group.

Variable	Total (*n* = 144)	High CXI group (*n* = 64)	Low CXI group (*n* = 80)	*p* Value
Gender (male/female)	84/60	39/25	45/35	0.5708
Age (y)^a^	69.3 ± 0.8	67.7 ± 1.2	70.6 ± 1.1	0.0774
BMI (kg/m^2^)^a^	22.1 ± 0.2	23.1 ± 0.4	21.4 ± 0.3	**0.0004**
DM (yes/no)	70/74	36/28	34/46	0.1009
Initial resectability (R/BR‐A/BR‐PV)	131/7/6	60/1/3	71/6/3	0.2527
Platelet (×10^4^/μL)^a^	22.6 ± 0.6	21.2 ± 1.0	23.7 ± 0.9	0.0578
Serum albumin (g/dL)^a^	3.9 ± 0.1	3.9 ± 0.1	3.8 ± 0.1	**0.0351**
Total bilirubin (mg/dL)^a^	0.8 ± 0.1	0.8 ± 0.1	0.8 ± 0.1	0.8763
*P*‐Amylase (U/L)^a^	50.8 ± 6.3	45.3 ± 9.5	55.1 ± 8.4	0.4411
NLR^a^	2.3 ± 0.1	1.58 ± 0.13	2.83 ± 0.11	**<0.0001**
PLR^a^	0.9 ± 0.1	0.63 ± 0.06	1.03 ± 0.05	**<0.0001**
LMR^a^	5.0 ± 0.3	6.28 ± 0.40	4.05 ± 0.36	**<0.0001**
mGPS^a^	0.25 ± 0.04	0.13 ± 0.06	0.35 ± 0.06	**0.0097**
TLC^a^	1591.2 ± 46.8	1821.4 ± 65.5	1407.0 ± 58.6	**<0.0001**
PNI^a^	46.5 ± 0.4	48.4 ± 0.6	45.0 ± 0.5	**<0.0001**
CONUT score^a^	1.9 ± 0.1	1.4 ± 0.2	2.3 ± 0.2	**0.0003**
CAR^a^	0.11 ± 0.02	0.079 ± 0.036	0.138 ± 0.032	0.2185
Preoperative chemotherapy (yes/no)	49/95	24/40	25/55	0.4315
Tumor location (Ph/Pb/Pt)	74/43/27	30/24/10	44/19/17	0.2916
CEA (ng/mL)^a^	5.3 ± 1.0	6.2 ± 1.6	4.5 ± 1.4	0.4310
CA19‐9 (U/mL)^a^	273.6 ± 47.2	188.5 ± 70.4	341.6 ± 63.0	0.1077
DUPAN‐2 (U/mL)^a^	435.8 ± 97.0	243.1 ± 144.0	622.4 ± 128.8	0.0516
SPan‐1 (U/mL)^a^	47.3 ± 7.4	30.4 ± 11.0	60.5 ± 9.8	0.0604
Operative time (min)^a^	324.5 ± 9.4	325.6 ± 14.1	323.5 ± 12.6	0.9125
Blood loss (g)^a^	308.3 ± 24.3	300.3 ± 36.6	314.6 ± 32.7	0.7716
Maximum tumor size (cm)^a^	2.8 ± 0.1	2.5 ± 0.2	3.1 ± 0.1	**0.0060**
Lymph node metastasis (yes/no)	90/54	33/31	57/23	**0.0153**
Differentiation (well, moderate/poorly)	62/82	30/34	32/48	0.4077
R0 resection (yes/no)	142/2	62/2	80/0	0.1113
pStage 1/2/3/4	16/91/25/12	10/41/8/5	6/50/17/7	0.2919
Postoperative complication CD (0–1/≥2)	82/62	33/31	49/31	0.2434
Adjuvant chemotherapy (yes/no)	118/26	50/14	68/12	0.2865

*Note*: Boldface *P* values are statistically significant.

Abbreviations: BMI, body mass index; BR‐A, borderline resectable with artery involvement; BR‐PV, borderline resectable with portal vein involvement; CA19‐9, carbohydrate antigen 19–9; CAR, C‐reactive protein‐to‐albumin ratio; CD, Clavien–Dindo classification; CEA, carcinoembryonic antigen; CONUT, Controlling Nutritional Status; CXI, cachexia index; DM, diabetes mellitus; LMR, lymphocyte‐to‐monocyte ratio; mGPS, modified Glasgow Prognostic Score; NLR, neutrophil‐to‐lymphocyte ratio; PLR, platelet‐to‐lymphocyte ratio; PNI, prognostic nutritional index; R, resectable; TLC, total lymphocyte count.

^a^ Data are expressed as mean ± standard error.

### 2.2 CXI

A hemogram and chemistry profile were conducted preoperatively. The CXI was calculated based on the preoperative SMI, albumin level, and NLR. The formula used to calculate the CXI was SMI/[height (m) × height (m)] × albumin/NLR. The SMI was calculated by measuring the major and minor diameters of the iliopsoas muscle at the third lumbar vertebra using preoperative computed tomography images. The SMI was calculated using the following formula: iliopsoas major axis (mm) × iliopsoas minor axis (mm) × π/100.^12^, ^13^ The CXI cutoff values were set with reference to the report by Tanji et al^13^ (22.90 for men and 16.58 for women).

### 2.3 Nutritional evaluation

Preoperative blood samples were obtained from all patients within 1 week before surgery. Blood tests in the preoperative treatment group also showed the results immediately before surgery, and the NLR, PLR, and lymphocyte‐to‐monocyte ratio (LMR) were calculated based on the preoperative blood values. The mGPS was calculated as previously described.^14^ Briefly, patients with both an elevated CRP concentration (>0.5 mg/dL) and low albumin concentration (<3.5 g/L) were assigned an mGPS of 2, patients with only one of these biochemical abnormalities were assigned an mGPS of 1, and patients with neither of these abnormalities were assigned an mGPS of 0. The total lymphocyte count was also evaluated. The PNI was calculated as follows: 10 × serum albumin concentration (g/dL) + 0.005 × lymphocyte count (cells/mm^3^). The CRP‐to‐albumin ratio was calculated by dividing CRP by serum albumin. The CONUT score was calculated using the serum albumin concentration, peripheral lymphocyte count, and total cholesterol concentration: (a) albumin concentrations of 3.5, 3.0–3.49, 2.5–2.99, and <2.5 g/dL were scored as 0, 2, 4, and 6 points, respectively; (b) total lymphocyte counts of 1600, 1200–1599, 800–1199, and <800 cells/mm^3^ were scored as 0, 1, 2, and 3 points, respectively; and (c) total cholesterol concentrations of 180, 140–179, 100–139, and <100 mg/dL were scored as 0, 1, 2, and 3 points, respectively. The CONUT score was defined as the sum of (a), (b), and (c).

### 2.4 Pathological evaluation

Pathological evaluation of surgical specimens was based on the tumor‐node‐metastasis (TNM) classification system of malignant tumors by the 7th edition of the classification of pancreatic carcinoma by the Japan Pancreas Society. Evaluation of the excised margin was performed as follows. R0 resection was defined as the absence of tumor cells on the pancreatic resection margin, nerve plexus dissection margin, portal vein dissection surface, posterior dissection surface, and bile duct dissection margin. By contrast, R1 resection was defined as the presence of tumor cells on these margins. In all cases, rapid intraoperative pathological diagnosis of the pancreatic resection margin was conducted, and additional resection was performed when tumor cells were still present on the stump surface.

### 2.5 Preoperative chemotherapy

According to the institutional protocols, 49 patients received modern combinatorial chemotherapy (including neoadjuvant therapy with either a 5‐fluorouracil‐based chemotherapy [FOLFIRINOX/FOLFOX] regimen or gemcitabine‐based [gemcitabine/nab‐paclitaxel] regimen or gemcitabine and S‐1 combination therapy) with or without dose modifications as deemed appropriate by the treating oncologists. Chemotherapy was administered on a 2‐ or 4‐week cycle depending on the specific regimen. Total cycles of chemotherapy were counted based on the total number of cycles administered during induction chemotherapy. Two selected patients received chemoradiotherapy as neoadjuvant therapy, and this was determined according to surgical/oncological recommendations in general based on the margin risk. In accordance with the institutional protocol, chemoradiotherapy consisted of photon/proton external beam therapy with a 50.4‐Gy dose delivered in 28 daily fractions over 5 weeks using 3D conformal or intensity‐modulated techniques with concurrent radiosensitizing chemotherapy.

### 2.6 Postoperative adjuvant chemotherapy

Postoperative adjuvant chemotherapy was introduced within 12 weeks after surgery and continued for 6 mo. The chemotherapy regimens included gemcitabine monotherapy, S‐1 monotherapy, gemcitabine and S‐1 combination therapy, or gemcitabine and nab‐paclitaxel combination therapy. No patients received postoperative radiation. After the operation, a blood examination including measurement of tumor markers (carcinoembryonic antigen, carbohydrate antigen 19–9 [CA19‐9], SPan‐1, and DUPAN‐2) and a computed tomography examination was performed every 3 mo for at least 5 y to screen for postoperative recurrence.

### 2.7 Propensity‐score matching analysis

The background factors of the patients were varied and did not represent a homogeneous population. Statistical methods to investigate the impact of cachexia on such a target patient population requires matching the background factors of the better and worse CXI groups as much as possible. Thus propensity‐score matching analysis was conducted for the patients. The cutoff value of CXI was determined by a receiver‐operating characteristic curve using the survival status at the 3‐y follow‐up in strata of sex. Two factors of maximum tumor size and lymph node metastasis of the metastatic lesion were selected for propensity‐score matching analysis. The caliber for the matching was set as 0.2.

### 2.8 Statistical analysis

The clinicopathological records of all 144 patients were collected and retrospectively reviewed. Differences between the high‐CXI group and low‐CXI group were assessed by the Mann–Whitney *U* test. Associations between variables were determined by Fisher's exact test or the χ^2^ test. Multivariate analysis of disease‐free survival (DFS) and overall survival (OS) after pancreatectomy for PDAC was performed using a Cox proportional regression model with a backward elimination stepwise approach. DFS and OS in both the preoperative treatment group and the nonpreoperative treatment group were calculated as years after resection. The survival curve was calculated using the Kaplan–Meier method with the log‐rank test. Statistical analyses were performed using JMP Pro 15.1 (SAS Institute, Cary, NC, USA). A *P*‐value of <0.05 was considered statistically significant.

## 3 RESULTS

### 3.1 Patient characteristics

The patients' characteristics are shown in Table 1. Their mean age was 69.3 y (range, 39–87 y). A preoperative nutritional assessment was performed using various indexes such as the NLR, PLR, and mGPS. The mean preoperative serum carcinoembryonic antigen and CA19‐9 concentrations were 5.3 ± 1.0 ng/mL and 273.6 ± 47.2 mAU/mL, respectively. Neoadjuvant chemotherapy was administered to 49 of 144 patients, and postoperative adjuvant chemotherapy was administered to 118 of 144 patients. The CXI was low in 80 (55.6%) of 144 patients. Clavien–Dindo grade >2 postoperative complications occurred in 62 (43.1%) of the 144 patients, including pancreatic fistula (*n* = 40), delayed gastric emptying (*n* = 12), bile leakage (*n* = 1), ascites (*n* = 7), deep vein thrombosis (*n* = 11), delirium (*n* = 9), and wound infection (*n* = 21).

### 3.2 Comparison between high‐CXI and low‐CXI groups

**FIGURE 1:**
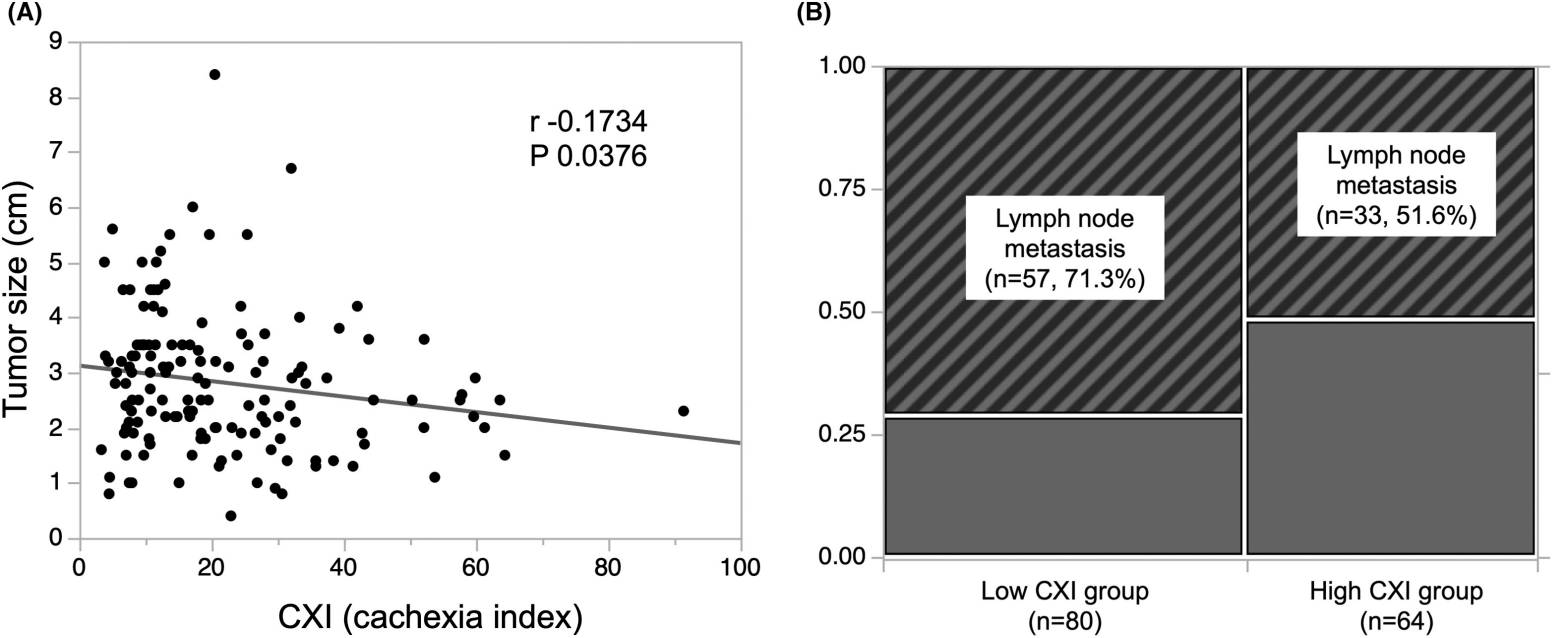
(A) Correlation between tumor size and CXI. (B) Relationship between lymph node metastasis and CXI. CXI, cachexia index.

Comparison of the high‐CXI group (*n* = 64) and low‐CXI group (*n* = 80) showed that the body mass index (BMI) and serum albumin concentration were significantly higher in the high‐CXI group than in the low‐CXI group (Table 1). Regarding nutritional factors, the high‐CXI group had a significantly lower NLR, PLR, mGPS, and CONUT score than the low‐CXI group, and the high‐CXI group had a significantly higher LMR, total lymphocyte count, and PNI than the low‐CXI group (Table 1). Additionally, the tumor size was significantly larger and lymph node metastasis was significantly more severe in the low‐CXI group than in the high‐CXI group (Table 1). There was no significant difference in the presence or absence of preoperative chemotherapy or postoperative chemotherapy between the two groups (Table 1). Moreover, a lower CXI was associated with a larger tumor size (*r* = −0.1734, *P =* 0.0376; Figure 1A), and the low‐CXI group (57/80, 71.3%) had significantly more lymph node metastasis than the high‐CXI group (33/64, 51.6%; *P =* 0.0153; Figure 1B).

### 3.3 Poor DFS and OS in low‐CXI group

**FIGURE 2:**
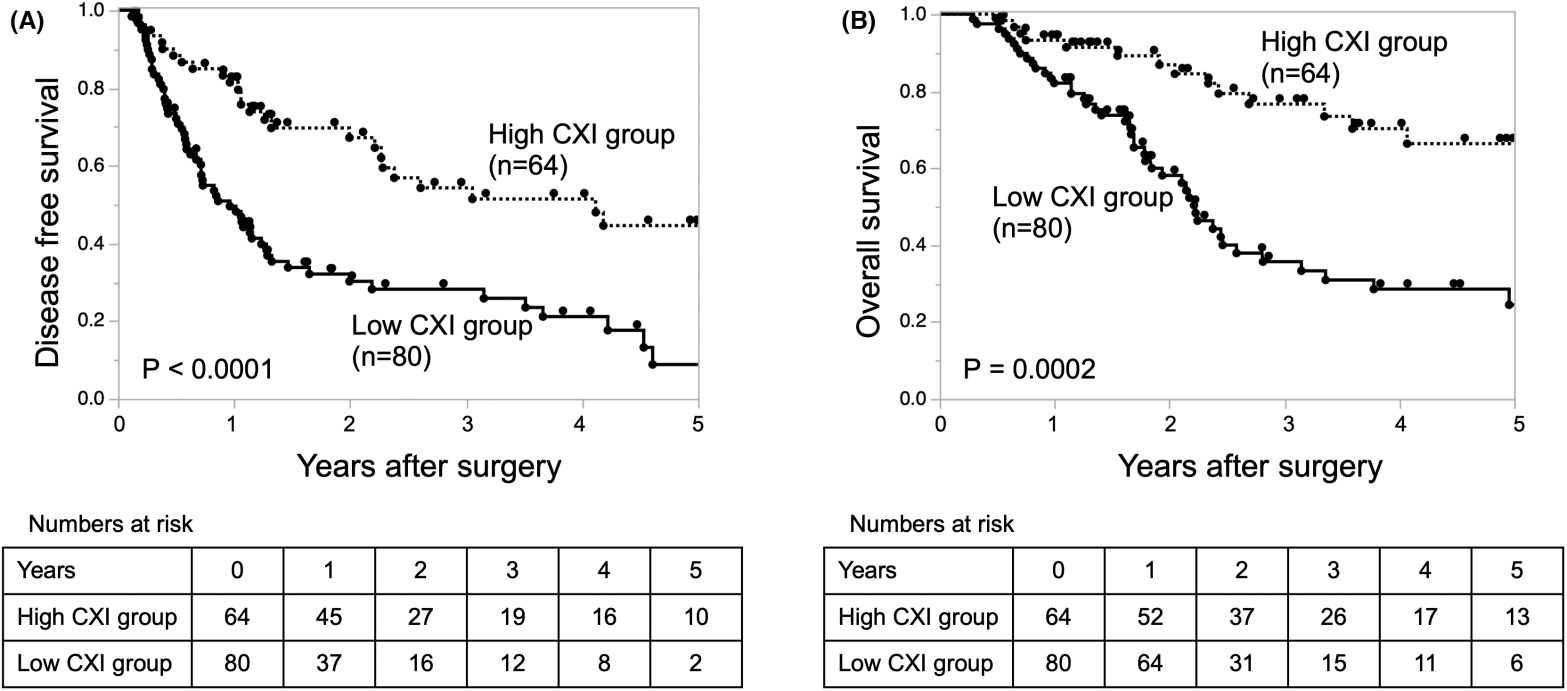
Kaplan–Meier analysis of (A) disease‐free survival and (B) overall survival of patients who underwent pancreatectomy for pancreatic ductal adenocarcinoma stratified by the CXI. CXI, cachexia index.

The low‐CXI group had significantly worse DFS than the high‐CXI group (*P <* 0.0001; Figure 2A). Additionally, the low‐CXI group had significantly worse OS than the high‐CXI group (*P =* 0.0002; Figure 2B). These results indicated that the low‐CXI group had a high recurrence rate and a poor prognosis in PDAC.

When compared among patients without lymph node metastasis (*n* = 54), patients with low CXI (*n* = 23) had a significantly worse prognosis than those with high CXI (*n* = 31; Figure S1A). Similarly, when compared among patients with small tumor size (<2.8 cm; *n* = 77), patients with low CXI (*n* = 34) had a significantly worse prognosis than those with high CXI (*n* = 43; Figure S1B).

### 3.4 Univariate and multivariate analyses of clinicopathological variables in relation to DFS after PDAC resection

The univariate analysis showed that a high CA19‐9 concentration (*P =* 0.0027), high DUPAN‐2 concentration (*P =* 0.0016), high SPan‐1 concentration (*P =* 0.0021), tumor size of >2.8 cm (*P =* 0.0001), lymph node metastasis (*P =* 0.0001), R1/2 resection (*P <* 0.0001), and inclusion in the low‐CXI group (*P <* 0.0001) were significantly associated with DFS after resection of PDAC (Table 2). The multivariate analysis confirmed that lymph node metastasis (hazard ratio [HR], 1.93; 95% confidence interval [CI]), 1.16–3.23; *P =* 0.0118), R1/2 resection (HR, 57.20; 95% CI, 9.39–348.30; *P <* 0.0001), and the low‐CXI group (HR, 2.10; 95% CI, 1.27–3.46; *P =* 0.0038) were independently associated with DFS in patients who underwent pancreatectomy for PDAC (Table 2).

**TABLE 2:** Univariate and multivariate Cox regression analyses of clinicopathological variables in relation to disease‐free survival after pancreatic resection for pancreatic ductal adenocarcinoma.

Variable	Univariate analysis			Multivariate analysis		
	HR	95% CI	*p* Value	HR	95% CI	*p* Value
Gender (male/female)	1.05	0.69–1.61	0.8254			
Age (≥69.3 vs < 69.3)	1.01	0.66–1.55	0.9487			
BMI (≥22.1 vs <22.1)	1.07	0.70–1.63	0.7713			
DM (yes/no)	1.34	0.88–2.06	0.1765			
Serum albumin (≥3.5 vs <3.5)	0.68	0.36–1.28	0.2318			
*P*‐Amylase (≥50.8 vs <50.8)	1.00	0.99–1.01	0.3041			
NLR (≥2.3 vs <2.3)	1.08	0.70–1.68	0.7195			
PLR (≥0.9 vs <0.9)	1.22	0.78–1.92	0.3758			
LMR (≥5.0 vs <5.0)	0.93	0.61–1.43	0.7492			
Preoperative chemotherapy (yes/no)	0.71	0.44–1.13	0.1500			
CEA (≥5.3 vs <5.3)	1.22	0.74–2.01	0.4363			
CA19‐9 (≥273.6 vs <273.6)	1.99	1.27–3.12	**0.0027**	1.48	0.87–2.51	0.1482
DUPAN‐2 (≥435.8 vs < 435.8)	2.12	1.33–3.39	**0.0016**	1.73	0.98–3.03	0.0569
SPan‐1 (≥47.3 vs < 47.3)	1.99	1.28–3.10	**0.0021**	1.03	0.56–1.90	0.9205
Tumor size (≥2.8 cm vs <2.8 cm)	2.37	1.53–3.67	**0.0001**	1.42	0.87–2.31	0.1619
Lymph node metastasis (yes/no)	**2.56**	**1.59–4.14**	**0.0001**	**1.93**	**1.16–3.23**	**0.0118**
Differentiation (poorly)	1.27	0.83–1.94	0.2785			
R0 resection (no/yes)	**42.19**	**7.60–234.3**	**<0.0001**	**57.20**	**9.39–348.30**	**<0.0001**
Adjuvant chemotherapy (yes/no)	0.90	0.51–1.60	0.7224			
CXI (low/high)	**2.56**	**1.61–4.07**	**<0.0001**	**2.10**	**1.27–3.46**	**0.0038**

*Note*: Boldface *P* values are statistically significant.

Abbreviations: BMI, body mass index; CA19‐9, carbohydrate antigen 19–9; CEA, carcinoembryonic antigen; CI, confidence interval; CXI, cachexia index; DM, diabetes mellitus; HR, hazard ratio; LMR, lymphocyte‐to‐monocyte ratio; NLR, neutrophil‐to‐lymphocyte ratio; PLR, platelet‐to‐lymphocyte ratio.

### 3.5 Univariate and multivariate analyses of clinicopathological variables in relation to OS after PDAC resection

The univariate analysis showed that a high CA19‐9 concentration (*P =* 0.0319), tumor size of >2.8 cm (*P =* 0.0376), lymph node metastasis (*P =* 0.0081), and inclusion in the low‐CXI group (*P <* 0.0001) were significantly associated with OS after resection of PDAC (Table 3). The multivariate analysis confirmed that inclusion in the low‐CXI group (HR, 3.14; 95% CI, 1.71–5.75; *P =* 0.0002) was independently associated with OS in patients who underwent pancreatectomy for PDAC (Table 3).

**TABLE 3:** Univariate and multivariate Cox regression analyses of clinicopathological variables in relation to overall survival after pancreatic resection for pancreatic ductal adenocarcinoma

Variable	Univariate analysis			Multivariate analysis		
	HR	95% CI	*p* Value	HR	95% CI	*p* Value
Gender (male/female)	1.19	0.71–1.98	0.5089			
Age (≥69.3 vs < 69.3)	1.16	0.70–1.92	0.5689			
BMI (≥22.1 vs <22.1)	1.50	0.90–2.48	0.1175			
DM (yes/no)	1.45	0.88–2.41	0.1458			
Serum albumin (≥3.5 vs <3.5)	0.87	0.43–1.76	0.6947			
*P*‐Amylase (≥50.8 vs <50.8)	1.38	0.81–2.35	0.2341			
NLR (≥2.3 vs <2.3)	1.20	0.72–2.00	0.4885			
PLR (≥0.9 vs <0.9)	1.46	0.87–2.44	0.1550			
LMR (≥5.0 vs <5.0)	0.80	0.48–1.33	0.3982			
Preoperative chemotherapy (yes/no)	0.55	0.30–1.01	0.0531			
CEA (≥5.3 vs <5.3)	1.14	0.58–2.26	0.6983			
CA19‐9 (≥273.6 vs < 273.6)	1.76	1.05–2.95	**0.0319**	1.48	0.87–2.54	0.1479
DUPAN‐2 (≥435.8 vs < 435.8)	1.36	0.75–2.48	0.3069			
SPan‐1 (≥47.3 vs < 47.3)	1.64	0.98–2.76	0.0604			
Tumor size (≥2.8 cm vs < 2.8 cm)	1.71	1.03–2.83	**0.0376**	1.08	0.63–1.86	0.7701
Lymph node metastasis (yes/no)	2.13	1.22–3.73	**0.0081**	1.56	0.86–2.84	0.1437
Differentiation (poorly)	1.28	0.77–2.11	0.3395			
R0 resection (no/yes)	2.79	0.38–20.58	0.3155			
Adjuvant chemotherapy (yes/no)	0.73	0.39–1.37	0.3273			
CXI (low/high)	**3.53**	**1.96–6.36**	**<0.0001**	**3.14**	**1.71–5.75**	**0.0002**

*Note*: Boldface *P* ‐values are statistically significant.

Abbreviations: BMI, body mass index; CA19‐9, carbohydrate antigen 19–9; CEA, carcinoembryonic antigen; CI, confidence interval; CXI, cachexia index; DM, diabetes mellitus; HR, hazard ratio; LMR, lymphocyte‐to‐monocyte ratio; NLR, neutrophil‐to‐lymphocyte ratio; PLR, platelet‐to‐lymphocyte ratio.

### 3.6 Propensity‐score matching analysis

**FIGURE 3:**
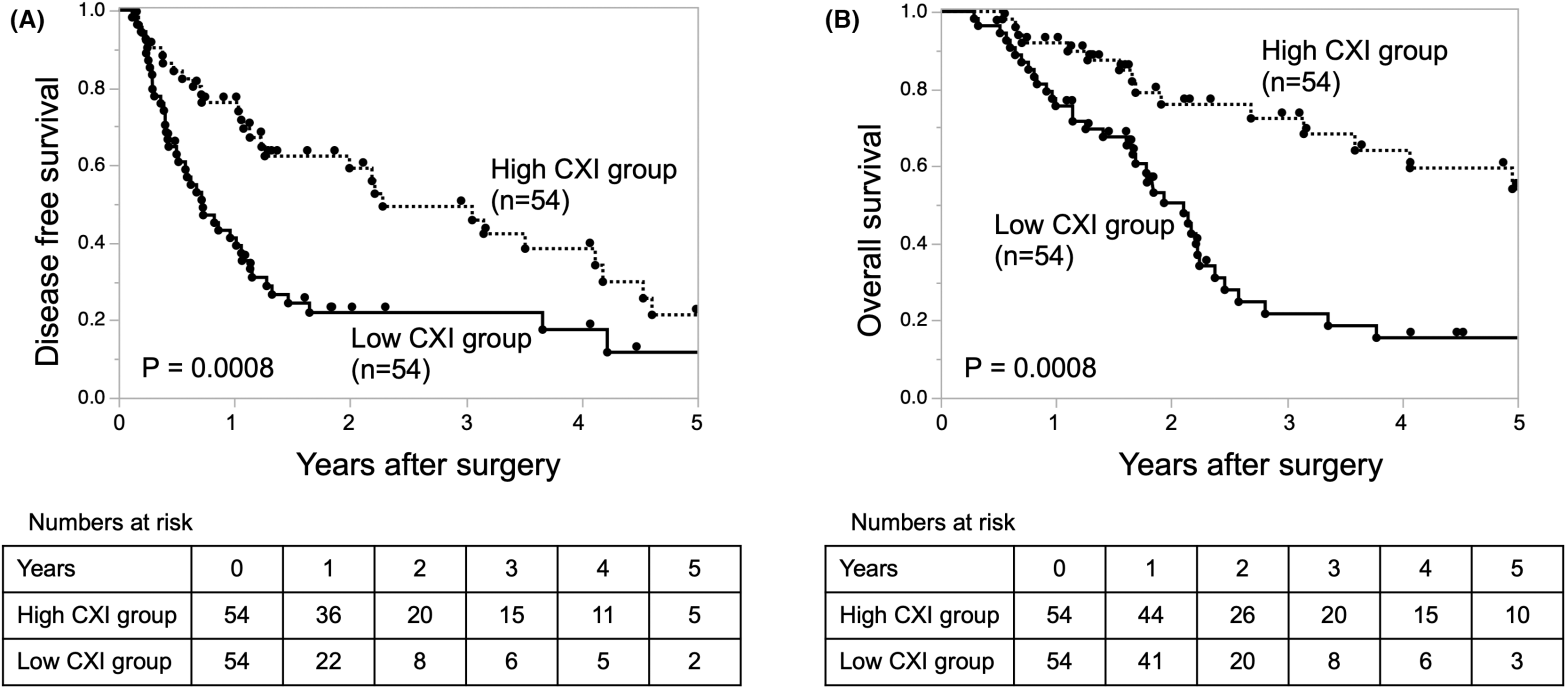
Kaplan–Meier analysis of (A) disease‐free survival and (B) overall survival of patients who underwent pancreatectomy for pancreatic ductal adenocarcinoma stratified by the CXI after propensity‐score matching. CXI, cachexia index.

The cutoff value of CXI was determined by a receiver‐operating characteristic curve using the survival status at the 3‐y follow‐up in strata of sex (18.37 for men and 7.96 for women). Propensity‐score matching analysis was conducted for all 144 PDAC patients; from 144 patients, 108 were selected, among whom 54 patients with high CXI and 54 patients with low CXI. The patients' characteristics and tumor characteristics after propensity‐score matching are shown in Table 4. There were no significant differences in characteristics observed between the two matched groups. The short‐term surgical outcomes of operative time, blood loss, and morbidity did not differ significantly between the two groups. The DFS curves and OS curves after propensity‐score matching are shown in Figure 3A,B. For both DFS and OS, the low CXI group had a significantly worse prognosis than the high CXI group.

**TABLE 4:** Patients' characteristics between the high‐CXI group and low‐CXI group after propensity‐score matching.

Variable	High CXI group (*n* = 54)	Low CXI group (*n* = 54)	*p* Value
Gender (male/female)	27/27	37/17	0.0502
Age (y)^a^	68.0 ± 1.4	71.0 ± 1.2	0.1240
DM (yes/no)	30/24	25/29	0.3358
Initial resectability (R/BR‐A/BR‐PV)	50/1/3	46/6/2	0.1396
Platelet (×10^4^/μL)^a^	21.4 ± 1.1	24.2 ± 1.2	0.0690
Total bilirubin (mg/dL)^a^	0.8 ± 0.1	0.8 ± 0.1	0.7475
*P*‐Amylase (U/L)^a^	41.5 ± 11.0	58.1 ± 10.6	0.2878
Preoperative chemotherapy (yes/no)	19/35	17/37	0.6831
Tumor location (Ph/Pb/Pt)	27/16/11	32/12/10	0.4945
CEA (ng/mL)^a^	4.9 ± 1.1	5.0 ± 1.3	0.9308
CA19‐9 (U/mL)^a^	354.1 ± 80.1	325.3 ± 87.1	0.8156
DUPAN‐2 (U/mL)^a^	366.9 ± 172.8	759.4 ± 178.4	0.1227
SPan‐1 (U/mL)^a^	45.2 ± 11.2	67.6 ± 13.7	0.2496
Operative time (min)^a^	320.4 ± 13.3	3330.1 ± 15.4	0.6545
Blood loss (g)^a^	285.9 ± 43.1	350.5 ± 41.8	0.2913
Maximum tumor size (cm)^a^	3.2 ± 0.2	3.0 ± 0.1	0.4092
Lymph node metastasis (yes/no)	37/17	37/17	1.0000
Differentiation (well, moderate/poorly)	25/29	22/32	0.5604
R0 resection (yes/no)	52/2	54/0	0.1534
pStage 1/2/3/4	4/32/9/9	6/35/11/2	0.1585
Postoperative complication CD (0–1/≥2)	33/21	35/19	0.6902
Adjuvant chemotherapy (yes/no)	43/11	45/9	0.6203

*Note*: Boldface *P* values are statistically significant.

Abbreviations: BR‐A, borderline resectable with artery involvement; BR‐PV, borderline resectable with portal vein involvement; CA19‐9, carbohydrate antigen 19–9; CD, Clavien–Dindo classification; CEA, carcinoembryonic antigen; CXI, cachexia index; DM, diabetes mellitus; R, resectable.

^a^ Data are expressed as mean ± standard error.

## 4 DISCUSSION

Prognostic factors other than the cancer stage, such as sarcopenia and nutritional predictors, have been studied in recent years. We demonstrated that the CXI, which is calculated using the SMI and NLR, was a prognostic predictor of postoperative outcomes in patients who underwent pancreatic resection for PDAC. This is the first study to show that a low CXI is closely related to poor clinical outcomes in patients with PDAC.

Cancer cachexia, a multifactorial syndrome primarily defined by the 2007 Cachexia Consensus Conference as a complex metabolic syndrome associated with underlying illness and characterized by loss of muscle with or without loss of fat mass, is associated with ~30% of cancer‐related deaths.^15^ Historically, cachexia was thought of as a syndrome of weight loss, anorexia, and fatigue, and a deteriorated life prognosis and quality of life among cancer patients. However, it was recently redefined precisely as the lean muscle mass loss associated with chronic illness and/or cancer.^16^ The European Palliative Care Research Collaborative in 2011 proposed a new definition and classification of cancer cachexia, which is divided into three levels: pre‐cachexia, cachexia, and refractory cachexia.^17^ It has been proposed that various factors and inflammatory cytokines produced by the tumor and host affect different cells of the body, such as muscle cells, hepatocytes, and adipocytes, causing metabolic abnormalities that lead to cachexia.^18^ For instance, inflammatory cytokines produced by tumor cells, such as tumor necrosis factor‐α and interleukin 6, are thought to contribute to muscle wasting and atrophy by inducing oxidative stress in skeletal muscles and activating muscle degradation pathways.^19^, ^20^, ^21^ Because cachexia has a complex pathophysiology, it is essential to have a composite index with which to measure ongoing cachexia rather than to use a single index such as the BMI.

The clinical characteristics of cancer cachexia are a poor nutritional status, systemic inflammation, and loss of muscle mass. Malnutrition is a frequent and serious problem in patients with various cancers, and it negatively affects many patients' quality of life and prognosis.^22^ Additionally, systemic inflammation, which is associated with a disturbance of various hematological components such as white blood cells (specifically neutrophils, lymphocytes, and monocytes) and platelets, plays a critical role in cancer progression.^23^ Clinical indicators of these features include the serum albumin concentration, SMI, and NLR. Each of these has been independently associated with a poor prognosis.^24^, ^25^, ^26^ Therefore, we decided that it would be beneficial to investigate whether the CXI, along with known factors, would serve as a better prognostic indicator for PDAC. Indeed, we found that the CXI was a better predictor of the prognosis than previously reported nutritional and inflammatory factors.

Compared with the high‐CXI group in this study, the low‐CXI group had more advanced‐age patients with a lower BMI, albumin concentration, LMR, total lymphocyte count, and PNI and a higher NLR, PLR, mGPS, and CONUT score (Table 1). Hence, we considered that the CXI reflects the status of the PNI, NLR, and PLR and facilitates comprehensive assessment of the nutritional status. However, we found no significant difference in the tumor marker of PDAC corelated to the CXI. Additionally, there were no significant differences in surgery‐related factors such as the operative time or intraoperative blood loss. This suggests that the CXI reflects the musculoskeletal status and that the nutritional status did not contribute to the content of the surgical procedure. Although one study showed that chemotherapy treatment was discontinued early and inpatient care was very common in patients with a low CXI because of treatment‐related toxicity,^27^ and although preoperative chemotherapy may induce skeletal muscle loss in patients with breast cancer,^28^ our study showed no difference in the proportion of patients who received neoadjuvant chemotherapy or postoperative adjuvant chemotherapy between the low‐CXI and high‐CXI groups. Also, in this study the CXI did not change significantly before and after neoadjuvant treatment (*P =* 0.8904). Therefore, we think that it is good to measure CXI before neoadjuvant treatment. If CXI measured before neoadjuvant treatment is low, rehabilitation and nutritional interventions may increase CXI before surgery. This may lead to an improved prognosis. However, there were no cases in which CXI was frequently measured before and after surgery in this study. We think that it is necessary to consider it in the future.

In the multivariate analysis, patients with a low CXI showed a poor prognosis with respect to both DFS and OS (Tables 2, 3). As a result, a low CXI was demonstrated to be an independent and significant risk factor for poor DFS and OS after pancreatic resection for PDAC. Moreover, patients with a low CXI had a larger tumor size and more lymph node metastasis than those with a high CXI (Figure 1A,B). These results suggest that prevention or improvement of CXI decline may improve long‐term outcomes by slowing tumor progression. Also, in patients without lymph node metastasis and with small tumor size, the low CXI group still showed a significant poor prognosis (Figure S1A,B). The results would support that CXI is a good predictor, not only as an oncological surrogate marker.

A number of reports have proposed that the CXI is a novel index for estimating cachexia that also correlates with the prognosis of malignant tumors such as colorectal liver metastasis, diffuse large B‐cell lymphoma, non‐Hodgkin's lymphoma, and advanced non‐small cell lung cancer (NSCLC).^12^, ^13^ Among patients with diffuse large B‐cell lymphoma, the low‐CXI group showed a predominant decrease in OS, and in patients with NSCLC and non‐Hodgkin's lymphoma, the low‐CXI group showed a predominant decrease in both OS and DFS; these findings are similar to those in patients with PDAC in the present study.^10^, ^12^, ^29^, ^30^ Therefore, the CXI may be associated with the treatment outcomes and prognosis of other cancers or diseases, and further research is needed.

A study is underway in Japan to improve cancer cachexia with the use of anamorelin, a ghrelin receptor agonist that can be administered orally.^31^ Ghrelin is an endogenous peptide hormone secreted mainly from the stomach, and it exhibits growth hormone secretion and appetite‐enhancing effects via growth hormone secretagogue receptor 1a.^32^ In Japan, the use of anamorelin for cancer cachexia was approved in 2020 for four types of cancers: PDAC, NSCLC, gastric cancer, and colorectal cancer.^33^ Further clinical studies using anamorelin or novel medications are expected to promote research on improving the CXI and the prognosis associated with it, as well as to improve research on the clinical outcomes of PDAC. Also, comprehensive treatment combined with nutritional therapy and exercise therapy is considered necessary for proper resolution.

Our study had two main limitations. First, it was a single‐center retrospective study involving a relatively small number of patients. Second, the retrospective nature of the study prevented comparison of blood data and imaging findings because of differences in the number of chemotherapy courses and the contents of chemotherapy. Therefore, a large‐scale prospective study is needed to validate the results.

The preoperative CXI was a reliable indicator to assess the DFS and OS after pancreatectomy for treatment of PDAC. The use of this index for clinical decision‐making will facilitate the development of a more accurate risk stratification system combined with tumor staging of PDAC. In conclusion, the CXI seems to be a useful predictor of poor DFS and OS after pancreatectomy for PDAC.

## AUTHOR CONTRIBUTIONS

T.S. participated in writing the article. T.S. and K.S. participated in the conception and design of the study. Y.M., E.O., and T.I. participated in the acquisition of data. Y.N., M.S., M.Y., and M.M. participated in the statistical analysis and interpretation of data. K.S. participated in reviewing the article. Y.T. participated in reviewing the article and providing final approval of the version to be submitted.

## FUNDING INFORMATION

No funding was received for this study.

## CONFLICT OF INTEREST STATEMENT

All authors declare no conflicts of interest for this article.

## ETHICS STATEMENT

This study protocol complied with the ethical guidelines of human clinical research established by the Japanese Ministry of Health, Labour and Welfare, as well as with the 1964 Helsinki Declaration and its later amendments. The study was approved by the Ethics and Indications Committee of the National Hospital Organization Kyushu Cancer Center (No. 2019–54). Informed consent was obtained from all individual participants included in the study.

## Supporting information


Figure S1.
