# Supplementary material for: Cachexia index as a prognostic predictor after resection of pancreatic ductal adenocarcinoma
Source: Ann Gastroenterol Surg. 2023 Apr 24;7(6):977–86. doi: 10.1002/ags3.12686 (PMC10623946; doi:10.1002/ags3.12686)
Supplement: Supplementary file 1 — Figure S1. [file AGS3-7-977-s001.zip › AGS3_12686_Supinfo.docx]

**Supplementary Information**

**Cachexia index as a prognostic predictor after resection of pancreatic ductal adenocarcinoma**

Tomonari Shimagaki^1^, Keishi Sugimachi^1^, Yohei Mano^1^, Emi Onishi^1^, Tomohiro Iguchi^1^, Yuichiro Nakashima^2^, Masahiko Sugiyama^2^, Manabu Yamamoto^2^, Masaru Morita^2^, Yasushi Toh^2^

^1^Department of Hepatobiliary and Pancreatic Surgery, National Hospital Organization Kyushu Cancer Center, Fukuoka 811-1395, Japan

^2^Department of Gastroenterological Surgery, National Hospital Organization Kyushu Cancer Center, Fukuoka 811-1395, Japan

**Supplementary Figure Legends**

**Supplementary Figure 1.** Kaplan–Meier analysis of overall survival of patients who underwent pancreatectomy for pancreatic ductal adenocarcinoma stratified by the CXI among (A) patients without lymph node metastasis (n = 54) and (B) patients with small tumor size (< 2.8cm) (n = 77).

CXI, cachexia index.
