# Supplementary figures and images for: Cachexia index as a prognostic predictor after resection of pancreatic ductal adenocarcinoma
Source: Ann Gastroenterol Surg. 2023 Apr 24;7(6):977–86. doi: 10.1002/ags3.12686 (PMC10623946; doi:10.1002/ags3.12686)

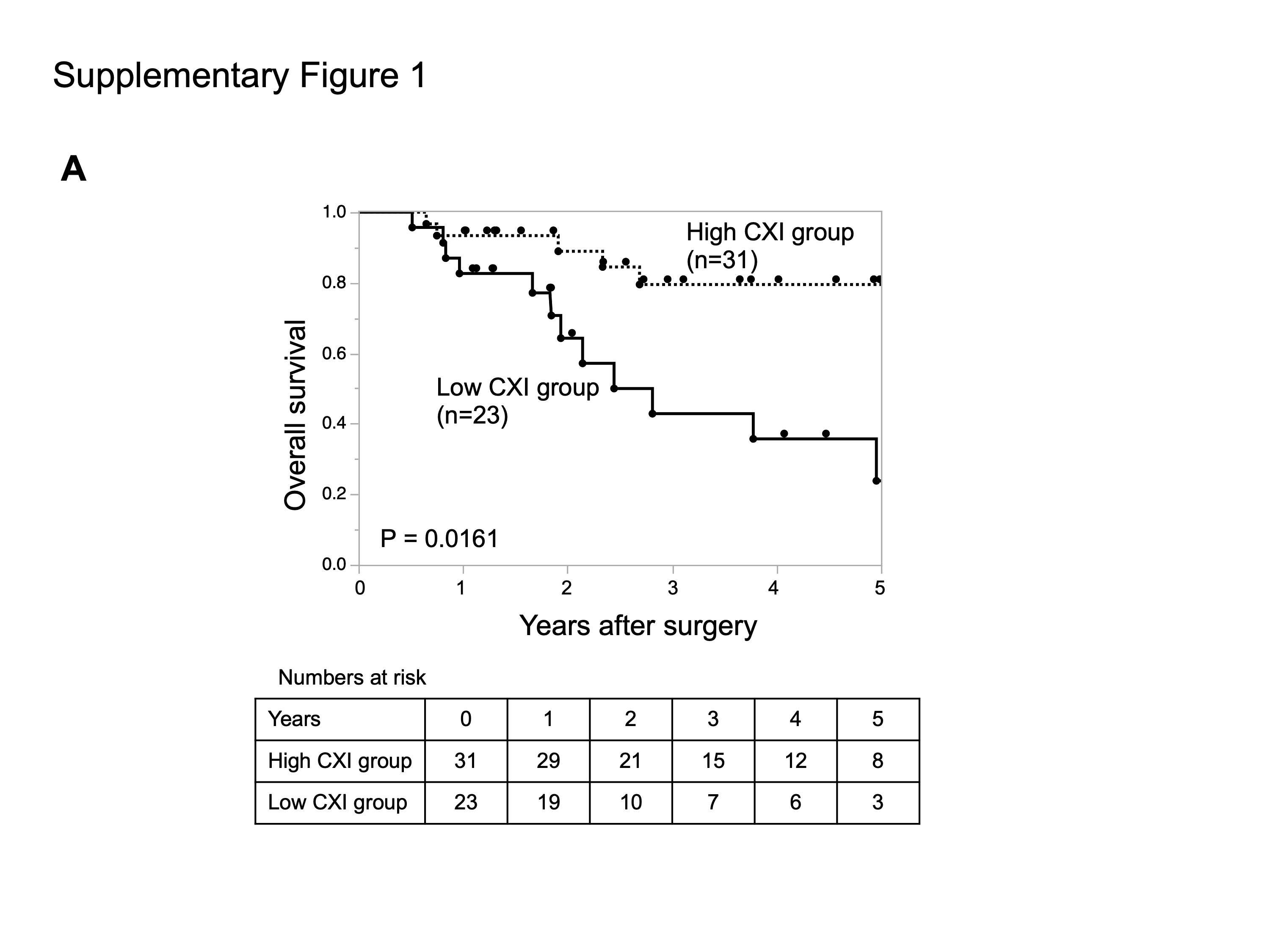

Supplement: Supplementary file 1 — Figure S1. [file AGS3-7-977-s001.zip › ags312686-sup-0001-FigureS1a.png]

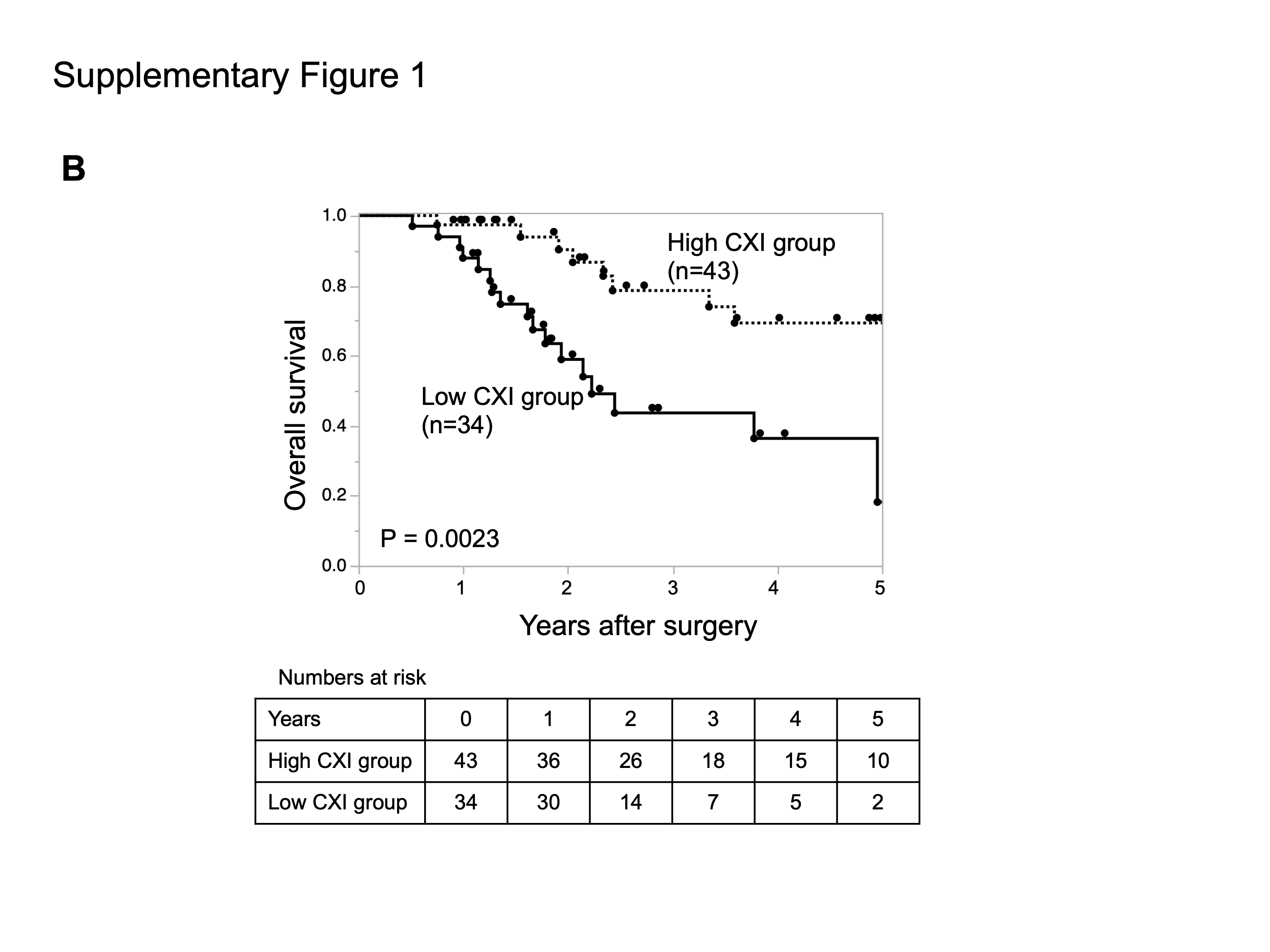

Supplement: Supplementary file 1 — Figure S1. [file AGS3-7-977-s001.zip › ags312686-sup-0001-FigureS1b.png]
